# Gut disruption impairs rehabilitation in patients curatively operated for pancreaticoduodenal cancer - a qualitative study

**DOI:** 10.1186/s12885-018-4933-1

**Published:** 2018-10-22

**Authors:** Kristine Elberg Dengsø, Tine Tjørnhøj-Thomsen, Susanne Oksbjerg Dalton, Bo Marcel Christensen, Jens Hillingsø, Thordis Thomsen

**Affiliations:** 1 Department of Gastrointestinal Surgery and Transplantation, Copenhagen University Hospital Rigshospitalet, 2100 Copenhagen, Denmark; 2 National Institute of Public Health, University of Southern Denmark, 1353 Copenhagen, Denmark; 3 The Danish Cancer Society Research Centre, 2100 Copenhagen, Denmark; 4 Abdominal Centre, Copenhagen University Hospital Rigshospitalet, Copenhagen, Denmark; 5 Department of Clinical Medicine, University of Copenhagen, Copenhagen, Denmark

**Keywords:** Cancer, Follow-up, Rehabilitation, HPB, Surgery, Symptoms, Qualitative

## Abstract

**Background:**

How patients recover and resume everyday life after curative hepato-pancreato-biliary (HPB) surgery with intestinal reconstruction has, to our knowledge, not previously been investigated. We wanted to explore the patient experience in order to develop our capability to support their rehabilitation and identify interventional gaps in the current post-surgical care of these patients. Therefore, the aim of the present study was to explore patients’ experiences of their gut, digestion, recovery and uptake of everyday life after HPB surgery with intestinal reconstruction.

**Methods:**

A qualitative explorative study with semi-structured interviews with 12 patients. We analysed data using qualitative content analysis with an inductive approach.

**Results:**

Two main themes with six sub-themes emerged from the analysis: 1. “Disrupted gut” covering the sub-themes: the weakened body; fighting cachexia; re-aligning to the altered body. 2. “Recovery work” with the sub-themes: the value of municipal rehabilitation programmes; reclaiming the sociality of meals; going back to work. The patients described overarching digestive changes, predominantly diarrhea and nausea. Diarrhea and nausea challenged rehabilitation efforts and limited patients’ participation in social activities. Patients toiled to regain strength and every-day life as it was before surgery. Current municipal rehabilitation programmes facilitated these efforts.

**Conclusions:**

The patients articulated an overarching experience of gut disruption, predominantly presenting as nausea, diarrhea and difficulty eating. This challenged their recovery work and uptake of every-day life. Specialised follow-up at expert centres might mitigate the sequelae of gut disruption after HPB surgery. We suggest that follow-up programmes systematically monitor the experienced symptoms of gut disruption with HPB-specific PROMS. Furthermore, research into the pathophysiology of cachexia and novel interventions for reducing cachexia and weakness after curative HPB surgery is relevant.

## Background

Standardised follow-up after curative surgery for cancers of the pancreas, duodenum or bile ducts has for years primarily focused on recurrence [1, 2]. Today, evidence suggests that standardised follow-up does not improve survival rates, but follow-up should rather focus on symptoms, nutrition, and psycho-social support to improve quality of life [1–3]. This is especially important in cancer diseases, where there is no curative treatment available on recurrence, but only a question of choosing the best palliative support for the individual patient [2, 3]. Patients with pancreas, duodenal and bile duct cancers often have a long history of disease before surgery. This means that they are vulnerable already at the time of diagnosis, i.e. they are often affected by hyperbilirubinemia, cachexia, weight loss, itching and so on [2, 3]. After major surgery with extensive resection and reconstruction of the gastrointestinal tract some symptoms, like cachexia, remain, and the operation itself can infer sequelae impacting on nutrition, well-being, and rehabilitation [4–6]. Traditionally, there has been great focus on physiological changes incurred by surgical resection and reconstruction, such as lack of vitamin B due to partial gastrectomy, endocrine function in case of diabetes, exocrine function due to decreased fat up-take, and reflux due to bile in the stomach. For example Gooden and White uncovered the difficulty of managing gut symptoms and complex dietary issues associated with pancreatic exocrine insufficiency in patients with pancreatic cancer [7]. At the same time, patients may also experience burdensome symptoms, pain and fundamental bodily changes which complicate rehabilitation and return to every-day life [5, 7–9].

Insight into experienced symptoms and challenges from the patients’ perspective is important for tailoring follow-up and supporting rehabilitation after surgery. The experience of patients after hepato-pancreato-biliary (HPB) surgery with intestinal reconstruction has, to our knowledge, however not been widely investigated. Consequently, we have limited knowledge of patients’ experiences of sequelae after curative surgery and adjuvant therapy for cancers of the pancreas, duodenum or bile ducts. Illuminating the patient experience may identify interventional gaps in the current post-surgical care of these patients and contribute to the patient-centeredness of future follow-up programmes as is also a priority issue for The Danish Health and Medicines Authority [1]. Therefore, the aim of the present study was to explore patients’ experiences of their gut, digestion, recovery and uptake of everyday life after HPB surgery with intestinal reconstruction.

## Methods

A qualitative explorative design with individual semi-structured interviews was adopted. The study is reported according to the COREQ (Consolidated Criteria for Reporting Qualitative Research Recommendations) recommendations for reporting of qualitative studies [10].

### Setting

The study was undertaken at a specialised referral centre in Denmark from November 2016 to February 2017. At this centre, patients with cancers of the pancreas, duodenum or bile ducts are offered the current standardised outpatient follow-up with consultations, blood tests and abdominal scans every third month for the first year and, after this, twice yearly. Follow-up commences when patients have undergone curative surgery and completed adjuvant chemotherapy. Patient pathways therefore differ somewhat, for example adjuvant therapy may for different reasons be delayed or paused intermittently while some patients do not require or want adjuvant chemotherapy. Patients therefore embark on the follow-up at varying time-points after surgery (between two to nine months).

In addition to the standardised follow up all patients are referred to rehabilitation programmes provided by local municipal centres. The municipal rehabilitation programmes vary slightly in duration and content but all focus on physical exercise, nutrition and offer healthy life style interventions. The majority of programmes last two months. If patients need extended rehabilitation they have access to continued support in the municipalities.

### Sample

Potentially eligible patients were identified by the patients’ assigned nurse in the outpatient clinic in connection with their first follow-up visit and asked if the first author (KD) could contact them with information about the study and an invitation to participate. We used purposive sampling as described by Patton [11] to sample participants according to gender and diagnosis. Data was collected until we deemed saturation was achieved [12]. Inclusion criteria were: ≥ 18 years old, histological and clinical diagnosis of cancer in the pancreas, duodenum or bile ducts and having commenced the outpatient follow-up programme. Interviews were conducted at a place of the patient’s choosing.

### Data collection and analysis

Data was collected through semi-structured interviews. We developed an interview guide as described by Johnson [13] inspired by our clinical knowledge and experience within the field [14, 15]. The interviews began with general small talk such as: “Thank you for letting me interview you”, in order to establish rapport. This was followed by more focused questions still allowing for an open approach and flexibility. The interview guide is presented in Table 1. Each patient completed a questionnaire to supply demographic information.

**Table 1:** Interview Guide

Themes	Examples of questions
Experiences of the beginning of the disease trajectory	Can you tell me how it all started? From the very beginning?
Experiences of everyday life problems in relation to disease	Please describe how an ordinary day unfolds for you now after your surgery
Experiences of return to home. Challenges, symptoms, digestion, working	How do you manage an ordinary day? Experiences of: Symptoms? Challenges? Needs?
Experiences of the ambulatory visit as an outpatient	What was your experience of your latest follow up visit? What did you talk about?
	Have you experienced any new or unresolved issues after your last outpatient visit?
General experience	Please describe follow-up support that has been helpful for you? Not helpful for you?

All interviews were digitally recorded and transcribed verbatim. We used NVivo Pro version 11 (QSR International Pty Ltd., Doncaster, Victoria, Australia) to manage the analysis of data. Qualitative content analysis with an inductive approach was used as outlined by Elo & Kyngas [16]. Inductive content analysis is appropriate when knowledge of an area is scarce [16]. The analysis entailed the following generic steps: 1. Reading the interviews to get a sense of whole, 2. Coding units of meaning and creating sub-themes, 3. Collapsing sub-themes into main themes [16]. Our aim was to analyse data at the manifest level, i.e. identify themes within the explicit or surface meanings of the data [16].

### Rigour

To ensure rigour we sought to satisfy the following four criteria: credibility, dependability, transferability and confirmability [12] matching the description from Shenton [17]. Credibility was obtained during interviews by KD by asking patients to validate her understanding of the patients’ experiences. Dependability was addressed during the analysis as three authors; (KD, TTT and TT) initially independently coded the data. Following this, KD, TTT and TT met to discuss outcomes from the independent coding process and to collapse sub-themes into main themes. To substantiate that the themes were empirically grounded, the authors (KD, TTT, TT) continuously moved back and forth between the original interview data, codes, sub-themes and themes. Final consensus for the analysis was reached through discussion in the entire author group in order to validate that the themes portrayed issues actually being expressed by the patients. The description of the patients’ characteristics and the study setting makes it possible for the reader to assess the transferability of the findings. Confirmability was obtained through the presentation of quotes supporting the themes and sub-themes [17, 18].

## Results

### Patients

Of fourteen eligible patients, twelve accepted participation. One patient declined, and one was excluded due to the presence of disseminated disease at the first outpatient visit. The mean age of the patients was 65 years (range 52–74), five were women, eight treated with chemotherapy, ten married and two lived alone. The median time since surgery was eight months (range 2–11 months). Interviews took place one week after the patients commenced follow-up. Eight patients were interviewed at home, three at the hospital, and one at work. The interviews lasted a median of 50 min (range 31–80).

### Themes

Two themes with six subthemes emerged; “Disrupted gut” and “Recovery work” (Table 2).

**Table 2:** Overview of themes and subthemes

Themes and sub-themes	Brief description of sub-themes	Number of patients experiencing the sub-themes
Disrupted gut		
The weakened body	Patients suffered from a range of gastrointestinal symptoms which negatively affected their digestion and prevented them from being fully active and participating in everyday activities and social life.	12
Fighting cachexia	Patients struggled to maintain or regain weight. They spent all their energy and time preparing and eating snacks.	10
Re-aligning to the altered body	The patients experienced bodily changes that they were forced to get accustomed to.	12
Recovery work		
The value of municipal rehabilitation programmes	Patients worked hard to regain strength and acknowledged the municipal health care centres for helping them do so.	10
Reclaiming the sociality of meals	Patients wanted to go out to restaurants and eat with friends; however, it was overwhelmingly burdensome to have to explain why they only could eat small portions or suddenly had to go to the toilet.	5
Going back to work	Actively employed patients wanted to go back to work as this symbolised normality life as it was before.	8

### Disrupted gut

All patients experienced gut disruption with nausea, belching, bloating and lack of appetite. Achieving sufficient nutritional intake was challenging and patients lost weight, and therefore lacked energy, physically and mentally. Weight loss altered the patients’ appearance and many subsequently struggled to re-align their image of themselves to the “new” emaciated and weakened body.

#### The weakened body

Gastrointestinal symptoms such as diarrhea, nausea, vomiting, fatigue, belching and bloating weakened patients, physically and mentally, and inhibited uptake of everyday activities. The disrupted gut and associated symptoms prevented patients from doing things they normally enjoyed. Diarrhea and nausea were described as the most disruptive and unpleasant symptoms. Some patients dared not leave their home or participate in other activities for fear of suddenly being sick, or not being able to reach a toilet in time.

*“My stomach was really bad after the surgery. You know, sometimes I didn’t even have time to get to the toilet. Not strange considering that it (the food) has to go through a completely different route, right. And I mean it (the stool) was literally water”. (Patient number 5).*

The symptoms often forced patients to change plans at very short notice.

*“And sometimes I have to go to the toilet NOW. And one day when I was on my way to the hospital I had to ask the driver to stop. And luckily there was a rest area and a toilet. And I remember I thought that if it hadn’t been there it would have been a disaster”. (Patient number 12).*

Some patients described a sense of social isolation. They could not leave their home for prolonged periods because they had to stay near a toilet due to the risk of erratic spouts of diarrhea.

*“Boy – I couldn’t even get up out of my chair and stand on my own two feet to begin with. I never thought I could be so weak. And getting to the first floor… that was almost… that was like running a marathon. So – uh – I stayed down here on the ground floor”. (Patient number 3).*

One patient described that she/he finally as a last resort started to tinker with the administration and dosing of Creon without consulting any health care professionals (HCPs).

*“Well, I’ve increased my dosage of Creon without asking anyone”. (Patient number 10).*

#### Fighting cachexia

Fighting to maintain or regain weight was an energy-consuming task for the patients. They spent a lot of time and energy, physically and mentally, preparing and forcing themselves to eat snacks throughout the day to get sufficient nutrients. They more or less focused all their waking hours and mental energy on trying to eat. The hospital provided patients with numerous information leaflets about nutrition, but they could not overcome reading.

*“I was given about a thousand different leaflets while I was in the hospital. You don’t get round to reading them. I’m probably a bit slow to get the hang of it – you know all the time it’s about snacking and I’m not used to eating snacks all the time. And if I have to, I get the feeling that I’m eating all day long. And that I have to prepare food all day long because all the time you can prepare this and prepare that. But I mean I’ve more or less just finished my breakfast when I have to start thinking about lunch”. (Patient number 10).*

Patients felt they were caught up in an ongoing battle against nausea, vomiting, lack of appetite, and extreme weight loss. Among those who tried to eat, some developed the dumping syndrome, which made them even sicker with hot flashes etc. Those experiencing the dumping syndrome furthermore worried whether this was something they would have to live with for the rest of their lives.

*“So they tried to get me to eat as much as possible but uhh… It was really hard for me and still is – unbelievably hard. Because I couldn’t hold very much food, and then I got dumping symptoms with hot flashes and I felt really terrible. And I threw up so many times… I had an appointment at the oncology department which I had to cancel because I simply couldn’t go anywhere (because of nausea)”. (Patient number 11).*

Some patients described losing their sense of taste or experiencing changes in their sense of taste which added to the difficulty of eating.

*“Uhh food tasted so bad – anyway that’s how I felt. I’m not choosy and I’ve never been choosy so it’s not because I didn’t like food, but all of a sudden I almost couldn’t… I more or less couldn’t eat anything”. (Patient number 2).*

#### Realigning to the altered body

All the patients worked hard to realign to their altered body. Their experiences of realigning varied with the majority of patients focusing on the overt, visible changes, following surgery and weight loss. A minority also reflected on having to realign to the internal, not-visible changes in their gastrointestinal tract, for example, the fact that they no longer had a duodenum or a gall bladder.

*“….to me everything has to do with my body. I mean the thought of not having a duodenum any longer, it’s missing, I think about that constantly”. (Patient number 1).*

The patients involuntarily compromised with aesthetics after surgery because bodily changes, drains and tubes prevented them from wearing the clothes they normally preferred to wear. Others described a visible bulging on their stomach; some constantly sensed the visible surgical scar while others felt their abdominal muscles were different after the surgery. Finally, patients had to realign their image of themselves according to how emaciated the body had become after surgery.

*“Everything has been moved around inside – so it’s not like it was and on top of that my stomach it’s… it’s all wrong.. I’ve got this bulge here…and bulges here as well…” (Patient number 2).*

*“…ughh I’ve struggled so much to eat so I could put on weight and I don’t think it looks good to be so skinny. I look terrible I mean you can see my ribs and everything. And when I got home I looked at myself in the mirror and saw the big scar after the surgery and thought to myself this can’t be you. It felt completely wrong”. (Patient number 11).*

### Recovery work

It was hard for patients to reclaim their everyday life and engage in social activities again postoperatively. They worked hard to pick up their former life and re-establish a sense of normality. Rehabilitation programmes provided by local municipalities were valuable facilitators of this process.

#### The value of municipal rehabilitation programmes

Patients overall expressed that they benefitted from participating in the rehabilitation programmes provided by municipal health care centres. They valued the ease of access to the municipal rehabilitation programmes, logistically and geographically. Specifically, they greatly appreciated not having to navigate between different geographical locations to talk to different HCPs about rehabilitation. Although one patient lacked high impact training in the municipal programme, the majority of patients regained strength and well-being through the municipal programmes.

*“What’s been great is that the physiotherapist, dietitian and psychologist work closely together. And they meet with each other. We meet with them”. (Patient number 6).*

*“ The physiotherapist who is my contact person said that I should join up and train at the same time as I was doing the municipal training. Because it’s a good supplement so… And well, at 3 pm I’m going over there (Fitness World) right. Twice a week. It’s great (laughter)”. (Patient number 5).*

#### Reclaiming the sociality of meals

The patients described the challenges of being able to enjoy a meal with others again after surgery, for example going out to restaurants or to friends’ homes. Eating normal size meal portions was physically impossible for them due to the extensive surgery. Because of this, some patients simply capitulated and stayed at home to avoid having to explain to friends and waiters why they could only eat small portions. For some patients, drinking alcohol was an integrated part of socialising with friends and family, and therefore not being able to drink alcohol was a major change they had to get accustomed to.

*“..It’s a bit hard when you go out to a restaurant to eat, I can tell you. Because you get all these questions….Why… don’t you like the food? Is there something wrong with the food? Just because you can’t eat up. Yes, and uhh you don’t really have the strength to go out because you’re thinking here it comes again, I have to answer all those questions again, again, again, again, right… And somehow I think it’s a bit annoying to have to explain each time, right…I also remember last summer we were hanging out in the city and the sun was shining. I was drinking a soda, and my friends were drinking cold beers. And I was just sitting there…right.” (Patient number 2).*

#### Going back to work

Going back to work was highlighted by patients as a significant marker of normality. Actively employed patients described how they missed colleagues and the normality and value work represented. Returning to work was of major importance for their uptake of everyday life.

*“ You feel that you’ve been put on hold, that you can just sit at home and watch others getting on with their lives and that’s not a good feeling. So I want to get back to work. Of course I can do some gardening and stuff like that but it’s not the same”. (Patient number 1).*

Patients described how important it was that the workplace allowed them time to recover gradually. This prevented feelings of guilt about being on sick leave or not being able to perform as before. Patients who experienced time to recover expressed less concern about their job situation and were able to concentrate their energy on recovering, physically and mentally.

*“It was nice to know so I also knew that I could take the time I needed. That my work place would be patient with me. I didn’t have to be concerned about that part. And as things went along I was on sick leave longer than I had thought just as my boss had said all along. I was gone for about 6-7 weeks before going back. And then I more or less didn’t do anything except be there for a few hours and then I went on my holidays and after that back to work for a few hours a day where I started to do a little work and then gradually I started doing more and more”. (Patient number 9).*

When returning to work, feeling well-taken care of by colleagues and not being burdened by too many tasks facilitated the transition back to “normality”. Some patients explicitly stated that they did not want any special attention from colleagues. They just wanted to go about their work as usual and not be asked about the cancer.

*“Well I called my work before going back and said that I’d like my boss to tell my colleagues (sobs) that it was alright to give me a hug and say hello but I didn’t want anyone asking me how I felt. Because, I just wanted to do my job. And feel normal again (sobs). And that also allows me to choose who I want to say what to”. (Patient number 7).*

## Discussion

We identified two key themes and six subthemes: The two key themes were: “Disrupted gut” and “Recovery work” and the six subthemes were: “The weakened body, Fighting cachexia, Re-aligning the altered body” and “The value of municipal rehabilitation programmes, Reclaiming the sociality of meals, Going back to work”. Our findings indicate that patients experienced major gastrointestinal disruption, with severe nausea, diarrhea and lack of appetite/ability to eat, and as a result, substantial weight loss and physical and mental weakness occurred. Weight loss altered the patients’ bodily appearance, forcing patients to re-align their image of themselves. They toiled to eat in order to regain strength and reclaim everyday life, physically, mentally, and socially. Municipal rehabilitation programmes were valuable facilitators of their rehabilitation and return to work, which was considered a significant step towards normality.

Diarrhea, belching, nausea and bloating were predominant symptoms that restricted nutritional intake resulting in weight loss, cachexia and physical and mental weakness. In lieu of a lack of effective treatments, patients described how they each individually tried to cope, and how their attempts to cope simultaneously drained them of energy and the ability/desire to participate in everyday social activities. Sandsund et al. similarly highlighted that persistent debility, weight loss, and fatigue resulted in physical and psychological sequelae that significantly restricted recovery in many patients [5]. Gooden et al. likewise found that the most debilitating symptoms in patients with pancreatic cancer were gas, belching and bloating, and pain/discomfort after eating and persistent diarrhea [7]. According to Bennani-Baiti et al. persistent cachexia leads to increased morbidity and mortality, but knowledge about the pathophysiology of cachexia and best treatment is sparse [19]. In relation to this, we found that cachexia not only affected physical recovery but also impacted negatively on the patients’ self-image and everyday lives, including companionship with family and friends, a finding also described by Reid et al. [20]. Gastrointestinal symptoms affecting physical and mental well-being appeared to be important to address at follow-up consultations as was ongoing counselling of patients about potential avenues for alleviating nausea and lack of appetite in order to prevent progressive weight loss. Ronga et al. stated that there is currently no effective treatment for cachexia in patients suffering from disseminated pancreatic cancer, and development of novel areas of therapeutic strategies is warranted. They argued that early intervention with nutritional, psychological and behavioural support is warranted along with nutritional supplementation and physical exercise [21].

To gain knowledge and develop and, subsequently, test clinically relevant interventions, we suggest that health professionals at each follow-up visit systematically elicit symptoms associated with gut disruption, and also patients’ lay experiences of successful or partial symptom alleviation. Development of valid HPB-specific patient-reported outcome measures (PROMS) would enable this. Monitoring of biomarkers of inflammation which is associated with cachexia might also be relevant to consider in future studies. Systematic prospective clinical collection of data on patient-reported outcome symptoms and experiences of symptom alleviation could provide key information to underpin relevant future follow-up interventions. If completed by patients prior to follow-up consultations, HPB-specific PROMS might further guide clinicians in focusing on the most urgent problems experienced by the patient. Expectation alignment prior to surgery regarding symptoms and avenues for symptom-alleviation after surgery after surgery may also prepare the ground for the patients’ postoperative recovery work.

Patients described that they were forced to re-align their self-image to a body transformed by weight loss and emaciation, and some patients reacted by staying away from social interaction. Hinsley and Hughes found that body-image was closely related to people’s construction of biography and self-identity, and that an altered body-image restricted socialisation patterns [14]. The patients in the present study wanted to interact socially and put in an enormous effort into eating and thereby take back their body and social life. Hinsley and Hughes reported similar findings and advocated that health care professionals include a social perspective in their approach to patients with cachexia [14].

Interestingly, although not outwardly visible, patients were acutely aware of and concerned with the changes imposed by surgery on their gastrointestinal tract. The experience of bodily changes after cancer has been widely investigated in patients with visible changes, for example in women after mastectomy [22]. We do not know whether the implications of living with non-visible, albeit seminal bodily changes, resemble those of living with visible bodily changes. This would be relevant to pursue further. Our findings moreover indicate that the bodily changes imposed by major resection of the gastrointestinal tract required substantial adaptation from patients. This suggests that it might be highly relevant to explore and discuss experiences of bodily changes with patients alongside supplemental medications for diabetes, malabsorption, reflux and B-vitamin deficiency.

Municipal rehabilitation programmes played an important role in facilitating the patients’ recovery work. In Denmark, municipal centres are responsible for rehabilitation after cancer treatment. Although the patients in the present study participated in slightly diverse rehabilitation programmes, their value to patients was apparent. These programmes are quite specific and inter-municipal collaboration has been set up in order to provide access to a wide range of interdisciplinary experts in order to ease the access of care.

Actively employed patients expressed the importance of going back to work. Work represented normality and companionship. A previous study found that specifically men had a strong desire to return to work while recovering from cancer [23]. Wells et al. reported that work enabled cancer survivors to re-gain a sense of normality, self-concept and identity; a sense of their former selves and a source of distraction [24]. As described by patients in the present study, workplace flexibility, for example in regard to working hours, work load and provision of assistance, was important for coping with fatigue and gradually taking on more tasks. Wells et al. highlight that cancer patients relied on information and guidance from health professionals for making decisions about returning to work [24]. Systematic reviews, however, reveal that effective vocational interventions are lacking in cancer care [25, 26]. Inclusion of vocational intervention in follow-up programmes is potentially highly relevant for general cancer rehabilitation and might include encouragement of patients to reflect on and discuss possibilities for gradual uptake of work with their workplace already before surgery.

In the short-term after HPB surgery with intestinal reconstruction, the disrupted gut was in the forefront. Nausea, diarrhea, belching, lack of appetite and substantial weight loss disturbed many facets of the patients’ everyday life. Bury described three aspects of disruption in the wake of unfolding chronic illness [27]. The first was disruption of taken-for-granted assumptions and behaviour and decisions about seeking help; the second involved a fundamental re-thinking of the person’s self-concept and biography; the third was the person’s response to disruption involving mobilisation of resources to adapt to the altered situation [27]. Analogous to this, patients in the current study appeared to be in the process of re-aligning their self-image to an altered body and to the uncertainties of the impact and course of the disease. Furthermore, they responded to the disruption of their gut by withdrawing from social gatherings to avoid embarrassing exposure to questions from strangers and acquaintances, social gatherings, which they nevertheless worked hard to take part in again [27]. An important finding was that actively engaging in municipal rehabilitation programmes and returning to work mediated patients’ mobilisation of resources and adaptation to the altered situation. Systematic collaboration between the hospital and municipal sectors in offering supportive rehabilitation programmes with inclusion of proactive vocational intervention might further support patients’ adaptation.

### Trustworthiness

The trustworthiness of our findings should be considered in terms of their credibility, transferability, dependability and confirmability [18]. To achieve credibility the interviews took place at a place chosen by the patients. Patients were sampled strategically to achieve rich and varied data. During interviews, the interviewer checked her understanding of the patients’ experiences [28], by asking the patients e.g. *“Is it correctly understood that….”?* or e.g. *“Is what you mean ….”?* Authors with different clinical backgrounds and distance to the research topic contributed to the analysis of data. This adds to the credibility and dependability of the findings. Confirmability was sought in the interaction between the presentation of the findings and the quotations illustrating them.

### Strengths and limitations

Strengths of this study were the rich data obtained from interviews and the analysis of data by three authors with different clinical backgrounds and distance to the phenomenon of interest. The patients had different diagnoses leading to the need for gastrointestinal resection and eight of twelve patients received chemotherapy after surgery. We consider that this heterogeneity increases the transferability of the findings. Sampling from different sites undertaking similar surgery might have allowed other nuances to emerge. However, the site from which patients were recruited is responsible for follow-up of half of patients undergoing HPB surgery in Denmark. Although we sampled from one site only, the patients attended slightly varying rehabilitation programmes, potentially making the transferability of the findings more uncertain.

## Conclusion

After HPB surgery with intestinal reconstruction, patients experienced overarching disruption of the gut, leading to weight loss, physical and mental weakness, difficulty partaking in social and rehabilitation activities, uptake of everyday life, and, for some, even social isolation. Prospective collection of clinical data on experienced symptoms and patient-initiated interventions for alleviating symptoms could perhaps inspire further development of follow-up programmes.

Municipal rehabilitation programmes contributed to the patients’ recovery work due to their proximity and the ease of access to interdisciplinary health professionals. Systematic collaboration between the hospital and municipal sectors in addressing the special needs of these patients might further support patients’ rehabilitation. Returning to work was important and we suggest inclusion of vocational guidance in future follow-up programmes.

## Abbreviations

COREQ: Consolidated Criteria for Reporting Qualitative Research Recommendations
HCP: Health Care Professionals
HPB: Hepato-pancreato-biliary
PROMS: Patient Reported Outcome Measures

## References

[1] Andersen, O., C. Iuul, and C. Erdland, Opfølgningsprogram for kræft i øvre mave-tarm T.D.H.a.M. Authority, Editor. 2015 The Danish Health and Medicine Authority www.sst.dk. p. 1–36. Accessed March 15 Mar 2015.

[2] Ducreux M (2015). Cancer of the pancreas: ESMO clinical practice guidelines for diagnosis, treatment and follow-up. Ann Oncol.

[3] Valle JW (2016). Biliary cancer: ESMO clinical practice guidelines for diagnosis, treatment and follow-up. Ann Oncol.

[4] Kapritsou M (2014). Fast-track recovery after major liver and pancreatic resection from the nursing point of view. Gastroenterol Nurs.

[5] Sandsund C (2013). Finding a new normal: a grounded theory study of rehabilitation after treatment for upper gastrointestinal or gynaecological cancers--the patient's perspective. Eur J Cancer Care (Engl).

[6] Hoybye MT (2008). Research in Danish cancer rehabilitation: social characteristics and late effects of cancer among participants in the FOCARE research project. Acta Oncol.

[7] Gooden HM, White KJ (2013). Pancreatic cancer and supportive care--pancreatic exocrine insufficiency negatively impacts on quality of life. Support Care Cancer.

[8] Elberg Dengso K (2017). Health-related quality of life and anxiety and depression in patients diagnosed with cholangiocarcinoma: a prospective cohort study. Acta Oncol.

[9] Torgerson S, Wiebe LA (2013). Supportive care of the patient with advanced pancreatic cancer. Oncology (Williston Park).

[10] Tong A, Sainsbury P, Craig J (2007). Consolidated criteria for reporting qualitative research (COREQ): a 32-item checklist for interviews and focus groups. Int J Qual Health Care.

[11] Patton MQ. Qualitative Research & Evaluation Methods - Integrating Theory and Practice. 4th ed. United States of America: Sage Publications Inc.; 2014. p. 832.

[12] Thorne S. Interpretive Description. Qualitative Reseach for Applied Pratice 2 ed. New York: Routledge Taylor and Francis group; 2016.

[13] Johnson JM. Handbook of interview research. Context & Method. 1st ed. California: Sage Publications, Inc; 2002.

[14] Hinsley R, Hughes R (2007). 'The reflections you get': an exploration of body image and cachexia. Int J Palliat Nurs.

[15] Faul LA (2012). Survivorship care planning in colorectal cancer: feedback from survivors & providers. J Psychosoc Oncol.

[16] Elo S, Kyngas H (2008). The qualitative content analysis process. J Adv Nurs.

[17] Shenton AK (2004). Strategies for ensuring trustworthiness in qualitative research projects. Educ Inform.

[18] Polit DF, Beck CT. Essentials of Nursing Research. Appraising Evidence for Nursing Practice. 8th ed. Philadelphia: The United States of America.

[19] Bennani-Baiti N, Walsh D (2009). What is cancer anorexia-cachexia syndrome? A historical perspective. J R Coll Physicians Edinb.

[20] Reid J (2009). Fighting over food: patient and family understanding of cancer cachexia. Oncol Nurs Forum.

[21] Ronga I (2014). Anorexia-cachexia syndrome in pancreatic cancer: recent advances and new pharmacological approach. Adv Med Sci.

[22] Piot-Ziegler C (2010). Mastectomy, body deconstruction, and impact on identity: a qualitative study. Br J Health Psychol.

[23] Handberg C, Nielsen CV, Lomborg K (2014). Men's reflections on participating in cancer rehabilitation: a systematic review of qualitative studies 2000-2013. Eur J Cancer Care (Engl).

[24] Wells M (2013). Supporting 'work-related goals' rather than 'return to work' after cancer? A systematic review and meta-synthesis of 25 qualitative studies. Psychooncology.

[25] Hoving JL, Broekhuizen ML, Frings-Dresen MH (2009). Return to work of breast cancer survivors: a systematic review of intervention studies. BMC Cancer.

[26] Tamminga SJ (2010). Return-to-work interventions integrated into cancer care: a systematic review. Occup Environ Med.

[27] Bury M (1982). Chronic illness as biographical disruption. Sociol Health Illn.

[28] Poth JWCCN. Qualitative Inquiry And Research Design. Choosing Among Five Approaches. Fourth ed. California: SAGE Publications; 2017. p. 488.

[29] World Medical Association, I. WMA Declaration of Helsinki - Ethical Principles for Medical Research Involving Human Subjects. 2017 2017. Available from: https://www.wma.net/policies-post/wma-declaration-of-helsinki-ethical-principles-for-medical-research-involving-human-subjects/. Accessed 16 Oct 2018.

